# Types of Adversity, Perceived Stressfulness and Resilience in Older Men and Women

**DOI:** 10.1155/jare/1018774

**Published:** 2026-04-01

**Authors:** Sini M. Stenroth, Markus J. Haapanen, Sini Siltanen, Pirjo Vuoskoski, Johan G. Eriksson, Mikaela B. von Bonsdorff

**Affiliations:** ^1^ Faculty of Sport and Health Sciences, Gerontology Research Center, University of Jyväskylä, Jyväskylä, Finland, jyu.fi; ^2^ Folkhälsan Research Center, Helsinki, Finland, folkhalsan.fi; ^3^ Department of General Practice and Primary Health Care, University of Helsinki and Helsinki University Hospital, Helsinki, Finland, helsinki.fi; ^4^ Faculty of Medicine, Centre for Health Services Research, Princess Alexandra Hospital, The University of Queensland, Woolloongabba, Queensland, Australia, health.qld.gov.au; ^5^ The University of Queensland, Australian Frailty Network, Woolloongabba, Queensland, Australia, health.qld.gov.au; ^6^ Faculty of Sport and Health Sciences, University of Jyväskylä, Jyväskylä, Finland, jyu.fi; ^7^ Department of Obstetrics and Gynecology and Human Potential Translational Research Programme, Yong Loo Lin School of Medicine, National University of Singapore, Singapore, nus.edu.sg

**Keywords:** adverse events, adversity, coping, resilience, stress

## Abstract

We investigated stressful adversities older adults face, examined whether older men and women experience them differently, assessed the stressfulness of these events and studied their relationship to resilience—the ability to overcome adversity. This cross‐sectional study included 1179 respondents from the Helsinki Birth Cohort Study, born between 1934 and 1944. At a mean age of 75 years, respondents reported the most stressful adversity in the past five years, its perceived stressfulness and resilience using the Hardy–Gill resilience scale. Adversities were coded using a qualitative content analysis. General linear modelling was used to examine the relationship between perceived stressfulness and resilience across types of adversity. We identified ten types of highly stressful adversities, with the five most common being personal illness (23.6%), illness (17.6%) or death (15.8%) of a close relative, relationship adversity (12.7%) and adversity related to living situations (8.6%). 4.8% of participants experienced ageing‐related changes as the most stressful adversities. Reported adversities were generally perceived as highly stressful, while resilience scores were intermediate. Men reported more self‐related adversity, perceived these as less stressful and showed better coping (a higher mean resilience scale score) than women after a highly stressful adversity. The identified highly stressful adversities included two previously unreported types related to the ageing process: ‘having to give up…’ (e.g., driving) and ‘health/functional decline’. Although the reported adversities were highly stressful, respondents coped well, suggesting a good capacity to manage adversity in later life.

## 1. Introduction

Ageing is often characterised by the occurrence of adversities, i.e., stressful life events [1], encompassing various types of social, physical and mental losses [2]. Previous studies have primarily used two types of approaches towards adversity in old age: some used interviews to identify adversities faced by older adults [3–5], while others [6–10] relied mostly on questionnaires such as the Elders Life Stress Inventory [11] or the Geriatric Life Event Scale [12], in which respondents could choose from predefined lists of adversities. This previous research has primarily relied on objective assessments of stress, often using preselected checklists of stressful life events, based on Holmes and Rahe’s [13] Social Readjustment Rating Scale. In contrast, the present study seeks to incorporate older adults’ perspectives by exploring their subjective perceptions of the most stressful adversity. This study enhances understanding of challenging adversities that shape older adults’ daily lives. These insights may help inform efforts to promote healthy ageing by identifying issues that influence functional capacity and independence in later life.

Because adversities are commonly referred to as ‘stressors’, they significantly contribute to the development of chronic stress in our lives. In the stress literature, these stressors are frequently categorised as life events, chronic strains and daily hassles [14]. Life events are regarded as short‐term, acute disruptions, whereas chronic strains are characterised by their persistent, ongoing nature and are often closely associated with close relationships, such as difficulties, conflicts or threats within marriage or parenthood [14, 15]. Daily hassles are minor, daily routine difficulties [16]. Overcoming these adversities, i.e., resilience, characterises healthy ageing [1]. To understand how individuals respond to adversity and demonstrate resilience, we need to consider the type, intensity, timing and duration of adversity [14, 17].

This study is based on Hobfoll’s Conservation of Resources (COR) theory [11]. COR theory [13, 14] emphasises that individuals need to ‘obtain, retain and protect resources’, and stress primarily arises when adversity surpasses these resources. Hobfoll’s theory identifies four kinds of resources: object resources (e.g., a car), condition resources (e.g., health, marriage), personal resources (intrinsic personal characteristics or skills, e.g., self‐esteem) and energy resources, which are valuable to obtain other resources (e.g., knowledge, money, time) [18, 19]. COR theory is shaped by three core principles introduced here shortly. The first principle emphasises ‘the primacy of resource loss’, which indicates that losing resources is more salient than gaining them [20]. The second principle concerns ‘resource investment’: resources should be invested when necessary to prevent resource loss, facilitate recovery or acquire additional resources [18, 20]. The third principle states that when resources are lost, processes for gaining resources activate and accelerate, and even small gains in the face of adversity may evoke hope for recovery and survival [18]. According to Hobfoll, events involving resource loss, such as the death of a loved one, divorce or retirement, are the most stressful [19].

We integrate the definition of resilience in successful ageing [21], which serves as the foundation for the Hardy–Gill resilience scale used in this study, with Hobfoll’s [18] definition of resilience within the COR theory. According to Rowe and Kahn’s theory of successful ageing [21], resilience should be defined in relation to the effects of a specific adversity and to the process of surviving it. Hobfoll [17] describes resilience as the capacity to endure the negative consequences of adversity while remaining active and engaged in daily routines. Thus, resilience manifests as a capacity to effectively manage the perceived consequences of the specific adversity and to recover and cope with them, while maintaining a committed, proactive and engaged stance [18, 21, 22]. Previous research suggests that older adults may have greater resilience than younger individuals [23, 24]. The literature also indicates that older men tend to experience less stress than women when confronted with adversities involving close relations [25, 26], such as a spouse’s illness [27]. However, the impact of these adversities on older men and women remains controversial, particularly regarding whether they use different coping strategies when dealing with adversity [24, 26, 28–30].

In this study, we were interested in the types of adversity older men and women encounter, how they perceive stress in the face of such adversities and how they cope with those they consider highly stressful. The study’s three primary questions and hypotheses were as follows: (1) What types of adversity do older men and women identify as the most stressful? We hypothesised, based on prior literature, that the most stressful adversities would be those related to one’s own health and the health of close others. (2) How stressful are these adversities perceived by older men and women? We hypothesised, based on earlier research, that perceptions of stress might differ between sexes. (3) How do older men and women cope with adversities that they consider highly stressful? We hypothesised that older men and women could cope effectively with adversity despite its high perceived stressfulness, and that coping with adversity (resilience) might differ between older men and women, as prior research has shown.

## 2. Materials and Methods

### 2.1. Study Design

This study used a mixed‐methods design, combining quantitative analyses of resilience and perceived stressfulness with a qualitative content analysis of respondents’ open‐ended descriptions of the most stressful adversity experienced during the past 5 years. The qualitative component was not used merely to label answers but to inductively construct a typology of adversity types, which then served as a key explanatory variable in the quantitative models. Integration of the two components occurred at the analysis stage, where the main adversity types derived from the qualitative analysis were used as categorical predictors in the general linear models.

### 2.2. Respondents

This cross‐sectional study utilised data from two data collection points in the Helsinki Birth Cohort Study (HBCS), which originally included 8760 men and women born at the Helsinki University Hospital, Finland, between 1934 and 1944 [31]. Briefly, HBCS is a longitudinal cohort study involving several subsequent clinical visits and a postal survey (see Supporting Table 1, Supporting Fig 1) [32, 33]. In the present study, we utilised data from the 2015 postal survey and from a clinical visit in 2017–2018. Both data collection points included the Hardy–Gill resilience scale, which asked about the most stressful adversity in the past five years, its perceived stressfulness and coping with this adversity. Among the 1153 respondents who completed the postal questionnaire in 2015, 966 (83.8%) answered the Hardy–Gill resilience scale, 6.3% reported not experiencing any stressful situations, and 9.9% did not respond or had incomplete responses. Similarly, of the 815 participants who attended the follow‐up clinical visit in 2017–2018, 96.9% respondents completed the Hardy–Gill resilience scale, 1.1% did not experience any stressful situation, and 2.1% did not respond or had incomplete responses. A total of 1179 respondents completed the Hardy–Gill resilience scale in 2015 and/or 2017–18, forming the sample for this study. Of the 1179 respondents, 49.4% completed the Hardy–Gill resilience scale at both data collection points. Since we were interested in the most stressful adversity experienced, we included only the Hardy–Gill resilience scale responses in which the respondent rated the identified adversity as more stressful.

Ethics Committee of the Hospital District of Helsinki and Uusimaa, along with the Ethics Committee of Epidemiology and Public Health, approved the HBCS. This study followed the European Parliament General Data Protection Regulation. Participants in the HBCS provided written consent before participating in the study.

### 2.3. Hardy–Gill Resilience Scale

*The most stressful adversity, perceived stressfulness and resilience* were assessed using the validated Hardy–Gill resilience scale [17], derived from the resilience module of the Asset and Health Dynamics (AHEAD) Study [34]. The first question on the Hardy–Gill resilience scale asks, in an open‐ended manner, about the most stressful adversity that occurred in the past 5 years. The second question asks about the perceived stressfulness of the adversity identified in the first question, using a visual analogue scale. The respondent places an X on a solid line ranging from ‘not at all’ (0 mm) to ‘extremely’ (140 mm). The distance from the starting point to the respondent’s mark is measured in millimetres, with higher values suggesting greater stressfulness.

Following this, the Hardy–Gill resilience scale [17] assesses resilience using nine structured questions. The first three questions address the perceived consequences of the adversity (e.g., ‘After this event, how much worse did you feel compared to before?’) and were scored on a 4‐point Likert scale from 0 (a great deal) to 3 (not at all). The remaining six multiple‐choice questions assess coping with the adversity, including two questions on each of the following: the time it took to feel better afterwards, changes in important activities and long‐lasting changes in feelings about life. Options for these six questions ranged from dichotomous (yes/no) to six categories (a few days, a few weeks, a few months, a year, more than a year or not better yet). Responses were paired and scored from 0 to 3 (e.g., Questions 4 and 5 were combined) to form a 6‐item resilience scale with a maximum score of 18. A higher score on the Hardy–Gill resilience scale indicates greater capacity to cope with adversity. Respondents were divided into three equal distribution‐based groups: low (0–9 points), intermediate (10–13) and high resilience (14–18).

### 2.4. Qualitative Data Analysis

Responses to the open‐ended question ‘Thinking back over the last 5 years, what has been the most stressful event in your life?’ from the Hardy–Gill resilience scale [17] formed the qualitative dataset. The responses consisted mainly of brief, factual descriptions, such as ‘illness of a spouse’. Given this structure, the purpose of the qualitative analysis was not to explore narrative meaning but to systematically categorise the surface‐level content of adversities to create a consistent, theoretically meaningful adversity typology for quantitative analysis. For respondents who listed multiple adversities (9.3%), only the first response was considered relevant and used for analysis, as respondents were instructed to answer with only one, the most stressful adversity over the last 5 years.

We employed a conventional content analysis approach [35], briefly described in Stenroth et al. [36]. Two researchers (SMS and KP) independently read all responses and coded each as a separate meaning unit. First, they generated initial descriptive codes capturing the semantic content of each adversity (e.g., ‘cancer’, ‘conflict’, ‘divorce’, ‘renovation’ or ‘injury’). Because most respondents did not specify details (e.g., type or duration of illness), codes were kept at a concrete, descriptive level to avoid overinterpretation. Second, the researchers inductively grouped similar codes into broader categories based on content similarity. Third, adversities were coded according to who was affected (e.g., ‘self’, ‘mother’, ‘child’ or ‘spouse’). Finally, adversities were classified as affecting the respondent versus someone else, in line with prior research showing that these distinctions may be meaningful [11].

**FIGURE 1:**
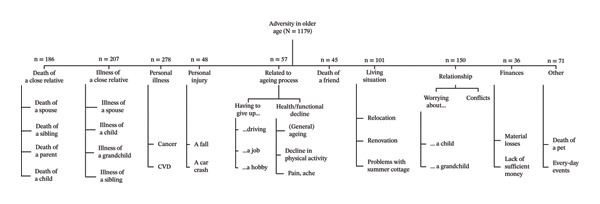
The most frequently reported adversity types among the Helsinki Birth Cohort Study respondents at a mean age of 75 years. Abbreviation: CVD = cardiovascular disease.

Coding discrepancies were discussed iteratively until full agreement was reached. Although no formal intercoded reliability coefficient was calculated due to brevity of responses, analytic rigour was ensured through repeated comparison sessions, joint code refinement and consensus‐building among three authors (SMS, KP and MBvB). This process yielded 10 overarching adversity types (with subtypes), as shown in Figure 1. These categories were subsequently used in the quantitative analyses as categorial predictors.

### 2.5. Statistical Analyses

All quantitative analyses were conducted using SPSS (Version 28). Baseline characteristics of the respondents were summarised using frequencies and percentages for categorical variables and means with standard deviations or medians with interquartile ranges for continuous variables, depending on distribution. Gender differences were examined using *χ* ^2^ tests for categorical variables and Mann–Whitney tests for continuous variables.

Because we found a significant interaction between gender and perceived stressfulness (*p* = 0.026), and prior research [24, 25, 28, 30] suggests gender differences in responses to adversity, analyses were conducted separately for men and women. Differences in perceived stressfulness and resilience across adversity types were analysed using univariate generalised linear models (GLMs). For these analyses, adversity subtypes were grouped into the five most common overarching adversity types. Adversity related to living situations was selected as the reference category because it was associated with the lowest stress levels and highest resilience scores in both genders. Models were first adjusted for age. Fully adjusted models additionally included socioeconomic status (SES), and physical and mental functioning summary scores (36‐Item Short Form Health Survey [SF‐36]). Effect sizes were calculated as partial eta squared for each association between adversity types and perceived stressfulness/resilience. Sensitivity analyses examined whether including the Beck Depression Inventory [37] instead of the SF‐36 mental functioning summary altered the findings.

### 2.6. Background Characteristics and Covariates

*Background characteristics and covariates* included age, gender, SES and physical and mental functioning (SF‐36). Age and SES were treated as time‐invariant covariates in our analyses (collected at the same occasion as the Hardy–Gill resilience scale), whereas SF‐36 scores were time‐varying. SES was categorised into upper officials (upper‐level employees with administrative, managerial, professional and related occupations), lower officials (lower‐level employees with administrative or clerical occupations), self‐employed and manual workers (e.g., workers in agriculture and forestry, manufacturing, distribution and service workers) according to the Statistics Finland classification [38]. Physical and mental functioning were assessed using the validated Finnish version of the SF‐36 [39]. Physical and mental component summaries were calculated from eight domains, which were standardised using the means and standard deviations of the US reference population and weighted using factor score coefficients from the respective population [40]. Higher scores imply better physical and mental functioning on a scale ranging from 0 to 100.

## 3. Results

### 3.1. The Types of Adversity and Background Characteristics

The content analysis yielded ten types of adversity: personal illness (*n* = 278, 23.6%), illness (*n* = 207, 17.6%) or death (*n* = 186, 15.8%) of a close relative; relationship adversity (*n* = 150, 12.7%); adversity related to living situations (*n* = 101, 8.6%); other adversity (*n* = 71, 6.0%); adversity related to the ageing process (*n* = 57, 4.8%); personal injury (*n* = 48, 4.1%); death of a friend (*n* = 45, 3.8%); and financial adversity (*n* = 36, 3.1%), as presented in Figure 1. The five most frequently reported adversities accounted for 78.2% of all events.

Regarding personal illness, respondents reported diagnoses including cancer and cardiovascular disease, though some did not specify their exact conditions. The most common responses concerning the death or illness of a close relative were the loss of a spouse or the spouse’s illness. Relationship adversity formed two subtypes: ‘worrying about…’ and ‘conflicts’. These subtypes addressed concerns and distress about close family members, such as children, grandchildren or spouses. Adversity related to living situations primarily involved relocating and renovations. The type ‘other adversity’ included a wide range of daily hassles to significant losses (e.g., death of a pet). For adversity related to the ageing process, two subtypes emerged. The first subtype, ‘having to give up…’ refers to situations in which respondents reported that age‐related changes had forced them to let go of cherished activities, e.g., hobbies. The second subtype, ‘health/functional decline’, pertained to general deterioration in health, which respondents described briefly, e.g., ‘declining health’ and ‘ageing in general’.

The characteristics of the study population are presented in Table 1. Among the study respondents, 44.0% (*n* = 519) were men, while 56.0% (*n* = 660) were women. The age range of the respondents was 70.5–83.7 years. Men had higher SES and better physical and mental functioning than women. Respondents rated adversities as highly stressful on a scale of 0 to 140 mm (mean 102.0, SD 32.1). However, despite the high perceived stressfulness of adversities, respondents coped well, i.e., adapted resiliently (mean 10.8, SD 4.0), indicating an intermediate level of resilience (low 0–9 points, intermediate 10–13 points and high resilience 14–18 points).

**TABLE 1:** Characteristics of the study population.

	Total *n* = 1179	Men *n* = 519	Women *n* = 660	*p*‐value
Age, years mean (SD)	74.96 (3.0)	74.91 (2.9)	75.1 (3.0)	0.684
Adult SES, *n* (%)				< 0.001
Upper official	202 (17.1)	124 (23.9)	78 (11.8)	
Lower official	533 (45.2)	149 (28.7)	384 (58.2)	
Self‐employed	114 (9.7)	54 (10.4)	60 (9.1)	
Manual worker	330 (28.0)	192 (37.0)	138 (20.9)	
Adversity, *n* (%)				< 0.001
Personal illness	278 (23.6)	156 (30.1)	122 (18.5)	< 0.001
Illness of a close relative	207 (17.6)	70 (13.5)	137 (20.8)	< 0.001
Death of a close relative	186 (15.8)	59 (11.4)	127 (19.2)	< 0.001
Relationship	150 (12.7)	52 (10.0)	98 (14.8)	0.014
Living situations	101 (8.6)	49 (9.4)	52 (7.9)	0.342
Other	71 (6.0)	44 (8.5)	27 (4.1)	0.002
Ageing process	57 (4.8)	32 (6.2)	25 (3.8)	0.059
Personal injury	48 (4.1)	21 (4.0)	27 (4.1)	0.969
Death of a friend	45 (3.8)	17 (3.3)	28 (4.2)	0.390
Finances	36 (3.1)	19 (3.7)	17 (2.6)	0.283
Perceived stressfulness, mean (SD)	102.0 (32.1)	90.2 (34.2)	103.0 (34.1)	< 0.001
Resilience score, mean (SD)	10.8 (4.0)	11.7 (3.7)	9.9 (4.1)	< 0.001
SF‐36, median (IQR)				
Physical functioning	47.4 (37.5–52.3)	48.3 (39.9–53.0)	46.1 (35.1–51.5)	< 0.001
Mental functioning	56.9 (50.0–60.4)	57.3 (52.2–60.4)	56.3 (48.3–60.3)	0.027

Abbreviations: IQR = interquartile range, SD = standard deviation, SES = socioeconomic status, SF‐36 = short form health survey.

Regarding adversities, men more frequently reported personal illnesses (30.1%, *n* = 156) compared to women (18.5%, *n* = 122; *p* < 0.001). Conversely, women reported experiencing illnesses (20.8%, *n* = 137% and 13.5%, *n* = 70; *p* < 0.001) and the deaths of close relatives (19.2%, *n* = 127% and 11.4%, *n* = 59; *p* < 0.001), and relationship adversity (14.8%, *n* = 98% and 10.0%, *n* = 52; *p* = 0.014) more often than men. Additionally, men reported lower levels of overall perceived stressfulness (mean 90.2, SD 34.2, and mean 103.0, SD 34.1) and higher resilience scores (mean 11.7, SD 3.7, and mean 9.9, SD 4.1) than women.

### 3.2. Perceived Stressfulness and Resilience According to Five Frequently Identified Types of Adversity

Men considered the illness of a close relative to be the most stressful type of adversity (Table 2), even after controlling for age, SES and functioning (regression coefficient = [B] 25.1; confidence intervals = [CI] 12.9, 37.3; *p* < 0.001). The second most stressful adversity for men was the death of a close relative in a fully adjusted model (B 15.7, CI 2.9, 28.4, *p* 0.016). Women identified the death of a close relative as the most stressful adversity (B 16.8, CI 8.3, 25.2, *p* < 0.001), with relationship adversity being the second most stressful adversity (B 9.6, CI 0.8, 18.4, *p* 0.032). The observed effect sizes were small (partial eta squared < 0.06). The fully adjusted model accounted for 11% of the variance in perceived stressfulness among men and 8% among women, indicating modest explanatory value, slightly higher for men than for women.

**TABLE 2:** Estimated marginal means (MM) and standard errors (SE) of *perceived stressfulness (scale from 0 to 140 mm)*, and unstandardised regression coefficients (B) and 95% confidence intervals (CI) according to five frequently identified adversity types stratified by gender.

	Model 1^1^		*p*	Fully adjusted^2^		*p*
	MM (SE)	B (95% CI)		MM (SE)	B (95% CI)	
Men						
Adversity related to living situations	80.3 (4.8)	ref.		82.1 (4.8)	ref.	
Personal illness	92.2 (2.7)	11.9 (1.0, 22.8)	0.033	88.4 (2.8)	6.2 (−4.9, 17.4)	0.272
Illness of a close relative	105.0 (4.0)	24.7 (12.4, 37.1)	< 0.001	107.2 (4.2)	25.1 (12.9, 37.3)	< 0.001
Death of a close relative	97.6 (4.4)	17.3 (4.4, 30.1)	0.009	97.8 (4.5)	15.7 (2.9, 28.4)	0.016
Relationship adversity	91.8 (4.7)	11.5 (−1.7, 24.7)	0.087	89.7 (4.8)	7.6 (−5.5, 20.7)	0.256
Women						
Adversity related to living situations	101.1 (3.7)	ref.		101.2 (3.7)	ref.	
Personal illness	108.2 (2.4)	7.1 (−1.5, 15.8)	0.107	107.3 (2.6)	6.0 (−2.5, 14.6)	0.168
Illness of a close relative	110.1 (2.3)	9.0 (0.5, 17.5)	0.038	110.2 (2.4)	9.0 (0.6, 17.3)	0.035
Death of a close relative	117.7 (2.4)	16.6 (8.0, 25.2)	< 0.001	117.9 (2.54)	16.8 (8.3, 25.2)	< 0.001
Relationship adversity	109.8 (2.7)	8.69 (−0.3, 17.7)	0.058	110.9 (2.8)	9.6 (0.8, 18.4)	0.032

^1^Model 1 adjusted for age.

^2^Fully adjusted model adjusted for age, SES and SF‐36 physical and mental functioning summary scores.

Both men and women reported their lowest resilience scores when facing the death of a close relative, also after adjustments for age, SES and functioning as shown in Table 3 (B −2.3, CI −3.5, −1.1, *p* < 0.001 and B −3.5, SE −4.6, −2.4, *p* < 0.001). Men reported the second lowest resilience scores when facing a close relative’s illness (B −1.7, CI −2.9, −0.6, *p* 0.003), while women reported the second lowest resilience scores when coping with personal illness (B −2.9, CI −4.0, −1.7, *p* < 0.001). The effect sizes were small (partial eta squared < 0.06) across all adversity types and both genders, indicating that individual adversity types contribute only modestly to resilience. The exception was the association between the death of a close relative and resilience among women, which showed a medium effect size. The fully adjusted model explained 32% of the variance in resilience scores for men and 29% for women.

**TABLE 3:** Estimated marginal means (MM) and standard errors (SE) of *resilience (low 0–9 points, intermediate 10–13 and high resilience 14–18)*, and unstandardised regression coefficients (B) and 95% confidence intervals (CI) according to five frequently identified adversity types stratified by gender.

	Model^1^		*p*	Fully adjusted^2^		*p*
	MM (SE)	B (95% CI)		MM (SE)	B (95% CI)	
Men						
Adversity related to living situations	13.5 (0.5)	ref.		13.2 (0.5)	ref.	
Personal illness	11.1 (0.3)	−2.4 (−3.6, −1.3)	< 0.001	11.7 (0.3)	−1.5 (−2.5, −0.4)	0.006
Illness of a close relative	12.0 (0.4)	−1.5 (−2.8, −1.2)	0.027	11.4 (0.4)	−1.7 (−2.9, −0.6)	0.003
Death of a close relative	11.0 (0.5)	−2.5 (−3.9, −1.1)	< 0.001	10.8 (0.4)	−2.3 (−3.5, −1.1)	< 0.001
Relationship adversity	11.8 (0.5)	−1.7 (−3.1, −0.3)	0.018	12.0 (0.5)	−1.2 (−2.4, 0)	0.057
Women						
Adversity related to living situations	12.4 (0.5)	ref.		12.2 (0.3)	ref.	
Personal illness	9.1 (0.3)	−3.2 (−4.5, −2.0)	< 0.001	9.3 (0.3)	−2.9 (−4.0, −1.7)	< 0.001
Illness of a close relative	10.9 (0.3)	−1.4 (−2.7, −0.2)	0.023	10.7 (0.3)	−1.5 (−2.6, −0.4)	0.008
Death of a close relative	8.9 (0.3)	−3.4 (−4.7, −2.2)	< 0.001	8.7 (0.3)	−3.5 (−4.6, −2.4)	< 0.001
Relationship adversity	10.2 (0.4)	−2.2 (−3.5, −0.9)	0.001	10.0 (0.4)	−2.2 (−3.4, −1.1)	< 0.001

^1^Model 1 adjusted for age.

^2^Fully adjusted model adjusted for age, SES and SF‐36 physical and mental functioning summary scores.

### 3.3. Sensitivity Analysis

In our sensitivity analysis (Tables S2 and S3), we replaced the SF‐36 mental functioning summary score with the Beck Depression Inventory, as prior studies indicate that depressive symptoms are associated with adversity and resilience [41, 42]. Among women, the association between the type of adversity and perceived stressfulness was no longer significant in cases of personal illness and relationship adversity. In contrast, no such differences were found among men. The findings regarding resilience remained similar for both genders. Sensitivity analyses did not significantly affect effect sizes.

## 4. Discussion

We investigated the most stressful adversity the respondents had experienced over the past 5 years at a mean age of 75 years and identified ten types of adversity. The five most common types—personal illness, the illness of a close relative, the death of a close relative, relationship adversity and adversity related to living situations—accounted for nearly 80% of all reported adversities. The qualitative approach also revealed that respondents experienced ageing‐related changes as stressful adversities. Although the reported adversities were highly stressful, the respondents effectively managed these challenges and recovered. This study expands understanding of older adults’ realities, which are often challenged by various adversities that can be viewed as resource losses, according to Hobfoll’s COR theory [19].

Older adults frequently acknowledge the challenges experienced by their close relatives, predominantly family members [5, 11, 12, 17, 43], a finding corroborated in the present study. For instance, Aldwin et al. [11] emphasise the importance of interpersonal relationships for older adults. In this study, half of the adversities identified as the most stressful over the past 5 years were events faced or affected by someone else—e.g., deaths, illnesses and worries about spouses, children and friends. Our findings also revealed that older men reported fewer adversities related to others, lower overall perceived stress and higher resilience scores than older women, consistent with previous research findings [21, 22, 26, 41–43]. In this study, older women identified the loss of a close relative, mostly a spouse, as particularly stressful and expressed low resilience scores after spousal loss. Adaptation to spousal loss might be easier for older men than for women, and several variables interact in coping trajectories; e.g., caring experience, education and personal strength have been suggested as factors that contribute to the adaptation process [43].

Previous research suggests that resilience to adversity may increase with age [23, 24] and that older adults respond resiliently to bereavement [14, 44]. These shifts towards older adults’ more resilient responses to adversity may reflect improved emotional regulation and more efficient coping strategies that conserve resources [45]. According to COR theory, accumulated resources across the life course can help alleviate adversity [19, 46, 47]. These resource gains are termed ‘resource caravans’, meaning that possessing one resource (i.e., self‐esteem) often leads to the possession of others [20]. Despite relatively resilient responses, our study supports Hobfoll’s [46] view that the loss of resources—such as marriage, financial stability or social networks—constitutes a salient stressor, particularly in older populations.

Identifying determinants of resilient responses seems complex. Bonanno et al. [48–50] suggest that the correlates of resilient outcomes are situational and vary over time; thus, it is impossible to find factors that are ‘beneficial in every situation or at every point in time’. This situational variability accounts for the small to modest effect sizes observed in perceived stress and resilience in the present study and contributes to modest gender differences in these scores. Effect sizes represent the proportion of variance in perceived stressfulness or resilience attributable to a given adversity type, after accounting for age, SES and functioning. Single adversity types explain only a modest proportion of multifactorial and complex stress responses. Due to variability in the timing, intensity and duration of adversities, along with differences in older adults’ resources for effective coping, small effect sizes are therefore comprehensible. Although our results may not be significant in real‐world contexts, they contribute to the existing evidence by indicating that types of adversity differ in perceived stress and that older men and women may respond differently to adversity. From a practical perspective, no single adversity type alone explains perceived stressfulness, resilience or gender differences. Rather, each adversity type makes a small but significant contribution, which should be taken into account when targeting interventions to address the negative health outcomes associated with adversity in older adults. Future studies should also adopt a life course approach that emphasises the variety, intensity and duration of adversities in old age, possible gender differences and the different resources older adults possess for coping with adversity.

### 4.1. Strengths and Limitations

The findings reported here provide finer grained information on the types of adversity older adults encounter. Using qualitative and quantitative methods, we analysed the most stressful adversity reported, perceived stressfulness and resilience among 1179 respondents in the HBCS. We complemented prior studies on the adversities faced by older adults. Identifying which types of adversity are perceived as highly stressful can deepen understanding of the challenges faced by the ageing population and provide new insights into the diversity of adversity types and how older men and women experience them. Moreover, knowledge of adversity related to the ageing process enhances understanding of the consequences of ageing, which can manifest as adversity faced by older adults.

This study has several limitations. First, the cross‐sectional design and focus on associations among adversity, perceived stressfulness and resilience limit causal inference. Therefore, we cannot determine the direction of causality for the observed associations. Second, we asked only about the most stressful adversity over the past 5 years to establish mutually exclusive types of adversity by using the Hardy–Gill resilience scale. Although identifying a single highly stressful adversity may have introduced less recall bias than using a checklist, we may have overlooked other significant information about these experiences, such as the duration and frequency of respondents’ encounters with them. This might have influenced the interpretation of our results and their generalisability. Third, data collection was conducted before the COVID‐19 outbreak. It should be noted that the pandemic may have affected how older adults perceive and cope with highly stressful adversities. Yet, we think the core of stressful adversity has remained unchanged—older adults still encounter the most frequently mentioned types of highly stressful adversity. Fourth, interrater reliability should be considered. Although two researchers coded the adversities, the coding was not independent, and we used only a loose coding frame based on previous literature. Fifth, our cohort comprised Caucasians; this should be considered when generalising the findings. The results of the current study may not be applicable to different geographical, cultural or social populations.

## 5. Conclusions

We identified ten types of highly stressful adversity experienced by older adults. The most frequently reported adversities were personal illness and the illness or death of a close relative, particularly a spouse. Moreover, we found two previously unreported types of adversity affecting older adults: adversity related to the ageing process and functional decline. Men reported more self‐related adversities, lower overall perceived stress and higher resilience scores than women. Overall, respondents demonstrated intermediate resilience, indicating that older adults can withstand highly stressful adversity. However, single adversities do not occur in a social vacuum. Rather, the adversities faced form a continuum of resource losses and gains spanning the life course from childhood to old age. Given the complexity and multifactorial nature of both perceived stress and resilience, we should further broaden our investigation to examine how adversities across the life course, from childhood to old age, associate with health outcomes in later life.

## Funding

This work was supported by the Juho Vainio Foundation, the Sigrid Jusélius Foundation and Samfundet Folkhälsan and Research Council of Finland (Grant Nos. 257239 and 349336). Open access publishing was facilitated by Jyvaskylan yliopisto, as part of the Wiley‐FinELib agreement.

## Ethics Statement

HBCS was approved by the Ethics Committee of Epidemiology and Public Health of the Hospital District of Helsinki and Uusimaa and the National Public Health Institute. The study followed the guidelines of the Declaration of Helsinki. All participants signed a written informed consent prior to participation.

## Conflicts of Interest

The authors declare no conflicts of interest.

## Supporting Information

Supporting Figure 1 Flowchart of the Helsinki Birth Cohort Study population. Data collection points used in this study are highlighted in yellow. Supporting Table 1 Clinical baseline characteristics (2001–2004) of the Helsinki Birth Cohort study population according to status in 2015 and 2017–18. Supporting Table 2 Estimated marginal means (MM) and standard errors (SE) of perceived stressfulness, and unstandardised regression coefficients (B) and 95% confidence intervals (CI) according to five frequently identified adversity types stratified by gender. The mental functioning summary scores replaced with scores from the Beck Depression Inventory. Supporting Table 3 Estimated marginal means (MM) and standard errors (SE) of resilience (low 0–9 points, intermediate 10–13 and high resilience 14–18), and unstandardised regression coefficients (B) and 95% confidence intervals (CI) according to five frequently identified adversity types stratified by gender. The mental functioning summary scores replaced with scores from the Beck Depression Inventory.

## Supporting information


**Supporting Information** Additional supporting information can be found online in the Supporting Information section.

## Data Availability

Pseudonymised data are available to external collaborators upon agreement on the terms of data use and publication of results.
