# Supplementary material for: Types of Adversity, Perceived Stressfulness and Resilience in Older Men and Women
Source: J Aging Res. 2026 Apr 1;2026:1018774. doi: 10.1155/jare/1018774 (PMC13045286; doi:10.1155/jare/1018774)
Supplement: Supplementary file 1 — Supporting Information Additional supporting information can be found online in the Supporting Information section. [file JARE-2026-1018774-s001.pdf]

Supplemental material

Types of adversity, perceived stressfulness, and resilience in older men and women

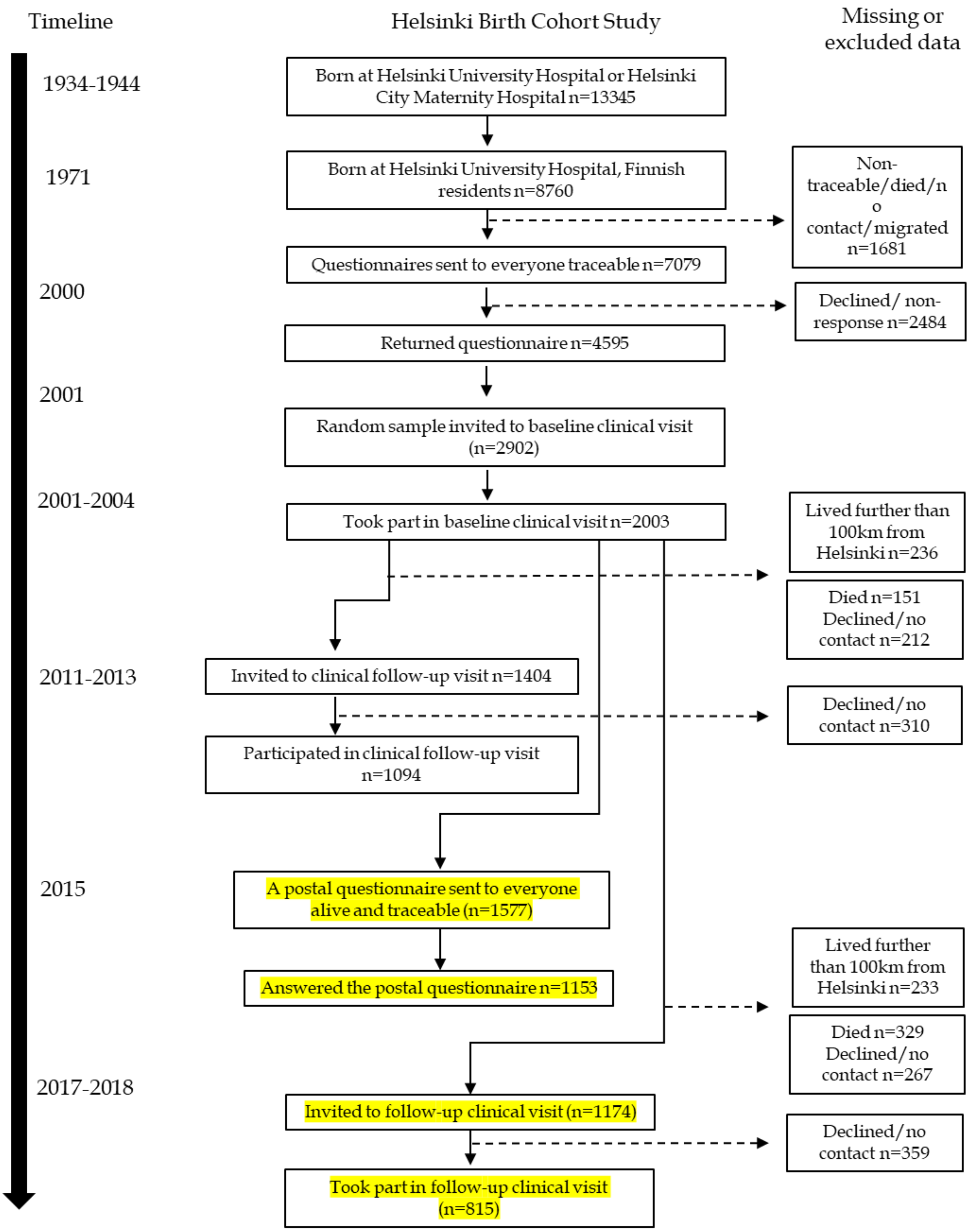

Supplemental Figure 1. Flowchart of the Helsinki Birth Cohort Study population. Data collection points used in this study are highlighted in yellow.

Supplemental Table 1 Clinical baseline characteristics (2001-2004) of the Helsinki Birth Cohort Study population according to status in 2015 and 2017-18.

	Participated		Declined/no contact		Died		p
**STATUS IN 2015**	**(n=1153)**		**(n=595)**		**(n=255)**		
Age at baseline, years mean (SD)	61.29	(2.8)	61.81	(3.1)	61.98	(3.1)	<.001
Men, n (%)	503	(43.63)	272	(45.71)	146	(57.25)	<.001
Adult SES, n (%)							<.001
Upper official	195	(16.9)	69	(11.7)	21	(8.6)	
Lower official	525	(45.5)	235	(39.7)	93	(38.0)	
Self-employed	105	(9.1)	50	(8.4)	31	(12.7)	
Manual worker	328	(28.4)	238	(40.2)	100	(40.8)	
SF-36, median (IQR)							
Physical functioning	51.29	(45.69-54.89)	49.67	(41.97-53.94)	47.67	(37.69-52.59)	<.001
Mental functioning	56.73	(51.29-59.77)	56.26	(50.79-59.55)	56.94	(51.39-60.22)	0.274
**STATUS IN 2017-18**	**(n=815)**		**(n=626)**[1]		**(n=329)**		
Age at baseline, years mean (SD)	61.12	(2.8)	61.85	(3.1)	62.09	(3.1)	<.001
Men, n (%)	357	(43.8)	277	(44.3)	190	(59.0)	
Adult SES, n (%)							<.001
Upper official	151	(18.5)	70	(11.2)	27	(8.5)	
Lower official	390	(47.9)	260	(41.7)	118	(37.0)	
Self-employed	80	(9.8)	38	(6.1)	38	(11.9)	
Manual worker	194	(23.8)	256	(41.0)	136	(42.6)	
SF-36, median (IQR)							
Physical functioning	51.90	(47.11-55.39)	49.86	(42.14-53.75)	48.30	(38.77-52.71)	<.001
Mental functioning	56.74	(51.82-59.63)	56.37	(50.39-59.61)	56.94	(51.21-60.05)	0.477

[1]The Helsinki birth cohort study members who declined or had no contact before the follow-up in 2017-18 (n=267) plus them who was invited to the follow-up but declined or had no contact (n=359), total of n=626

Loss to follow-up: The Helsinki birth cohort study members who did not participate in 2015 and 2017-18 were slightly older and had lower socioeconomic status and physical functioning

Supplemental Table 2 Estimated marginal means (MM) and standard errors (SE) of *perceived stressfulness (scale from 0 to 140mm)*, and unstandardized regression coefficients (B) and 95% confidence intervals (CI) according to five frequently identified adversity types stratified by gender. The mental functioning summary scores replaced with scores from the Beck Depression Inventory.

	**Fully adjusted**[1]		
	**MM (SE)**	**B (95% CI)**	**p**
**Men**			
Adversity related to living situations	82.1 (4.8)	ref.	
Personal illness	88.2 (3.0)	6.1 (-5.2, 17.4)	.291
Illness of a close relative	107.7 (4.2)	25.6 (13.3, 37.9)	<.001
Death of a close relative	99.9 (4.7)	17.8 (4.8, 30.8)	.007
Relationship adversity	89.0 (5.1)	6.9 (-6.6, 20.5)	.315
**Women**			
Adversity related to living situations	102.8 (4.0)	ref.	
Personal illness	108.0 (2.7)	5.2 (-3.9, 14.4)	.260
Illness of a close relative	109.4 (2.5)	6.6 (-2.3, 15.5)	.148
Death of a close relative	116.7 (2.7)	13.9 (4.8, 22.9)	.003
Relationship adversity	110.4 (3.0)	7.6 (-1.9, 17.)	.117

[1]Fully adjusted model adjusted for age, SES and SF-36 physical summary scores and scores from *the Beck Depression Inventory*.

Supplemental table 3 Estimated marginal means (MM) and standard errors (SE) of *resilience (low 0–9 points, intermediate 10–13 and high resilience 14–18)*, and regression coefficients (B) and 95% confidence intervals (CI) according to five frequently identified adversity types stratified by gender. The mental functioning summary scores replaced with scores from the Beck Depression Inventory.

	**Fully adjusted[1]**		
	**MM (SE)**	**B (95% CI)**	**p**
**Men**			
Adversity related to living situations	13.2 (.5)	ref.	
Personal illness	11.7 (.3)	-1.5 (-2.6, -.4)	.006
Illness of a close relative	11.5 (.4)	-1.7 (-2.9, -.6)	.003
Death of a close relative	11.0 (.5)	-2.2 (-3.5, -1.0)	<.001
Relationship adversity	12.0 (.5)	-1.3 (-2.6, 0.)	.050
**Women**			
Adversity related to living situations	12.4 (.5)	ref.	
Personal illness	9.2 (.4)	-3.3 (-4.5, -2.1)	<.001
Illness of a close relative	10.7 (.4)	-1.7 (-2.9, -0.6)	.004
Death of a close relative	8.9 (.4)	-3.5 (-4.7, -2.3)	<.001
Relationship adversity	10.2 (.4)	-2.2 (-3.4, -0.9)	<.001

[1]Fully adjusted model adjusted for age, SES and SF-36 physical summary scores and scores from *the Beck Depression Inventory*.
